# Supplementary material for: Permanent ferroelectric retention of BiFeO3 mesocrystal
Source: Nat Commun. 2016 Oct 26;7:13199. doi: 10.1038/ncomms13199 (PMC5095170; doi:10.1038/ncomms13199)
Supplement: Supplementary Information — Supplementary Figures 1-7, Supplementary Notes 1-5 and Supplementary References [file ncomms13199-s1.pdf]

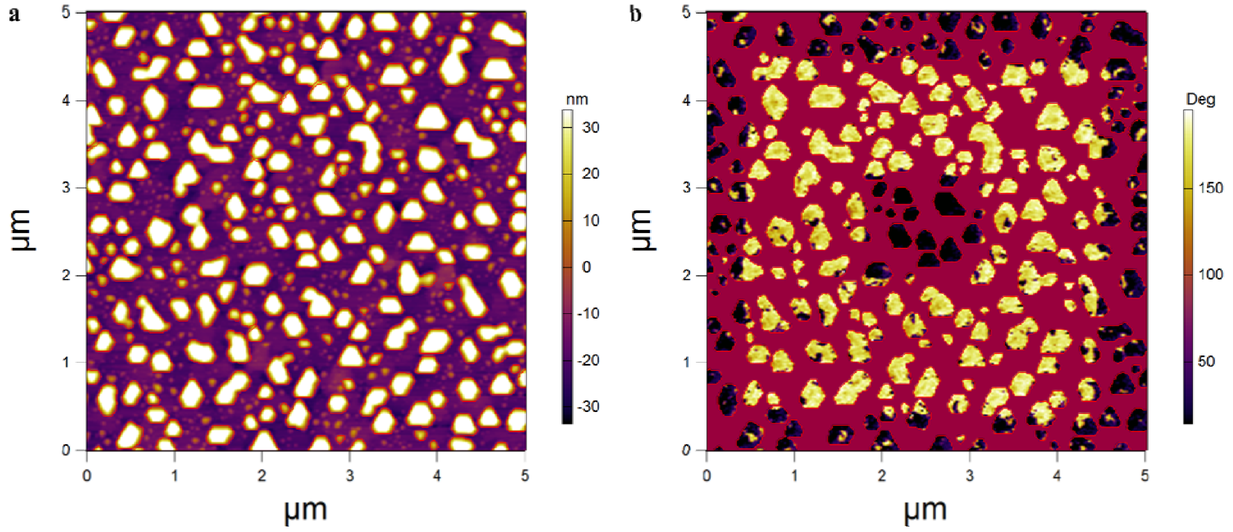

Supplementary Figure 1 Polarization switching of BFO mesocrystal. a. The topography and **b**. the corresponding PFM of BFO mesocrystal of 80 nm thick. The distribution of BFO mesocrystal in the topography image is depicted and overlapped on the PFM image.

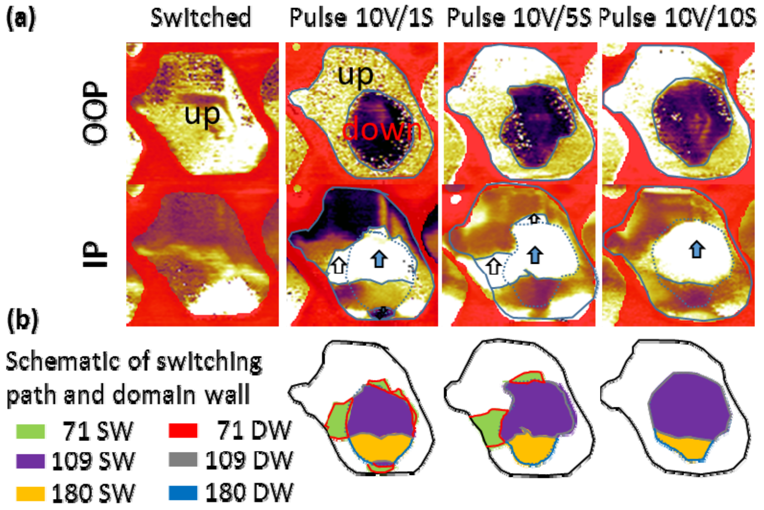

Supplementary Figure 2 Identification of the switching path and domain wall. a. The out-of-plane (OP) and in-plane (IP) phase images of BFO mesocrystal before and after hit by a voltage pulse. **b.** The schematics of the combination of OP and IP phase images in **a**. The arrows represent the orientation of polarization.

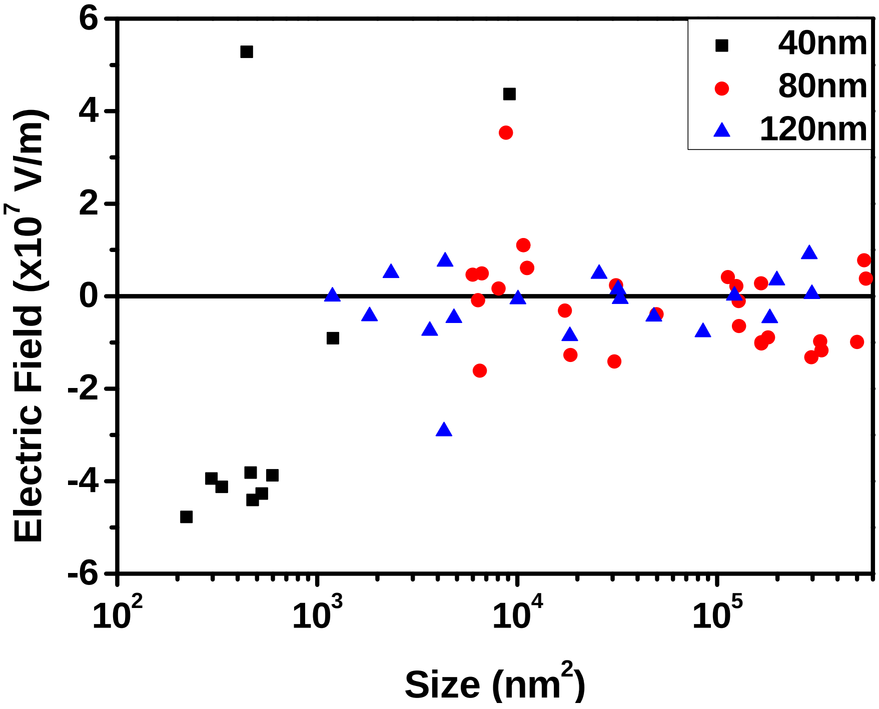

Supplementary Figure 3 Distribution of the center of hysteresis loops with mesocrystal size. The distribution of the deviation of the center of the hysteresis loop of the three thicknesses systems with different $BiFeO_3$ mesocrystal size. The center of the hysteresis loop is calculated as $[(+E_{\text{coercive field}}) + (-E_{\text{coercive field}})]/2$, $E_{\text{coercive field}}$: the value of coercive field.

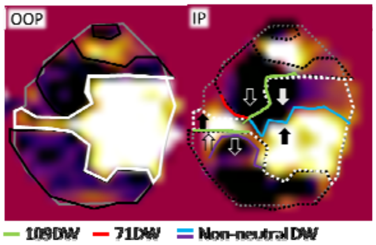

Supplementary Figure 4 Appearance of different domain wall in BOF mesocrystal during the relaxation precess. The OOP and IP PFM images of BFO nanocrystal. The arrow is the orientation of polarization.

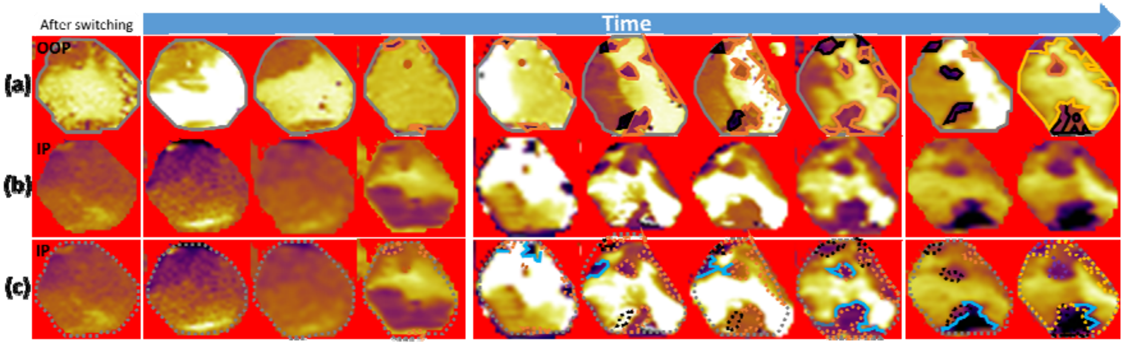

Supplementary Figure 5 The formation of domain walls during the relaxation process. The change of polarization of BFO nanocrystal during the relaxation. **a.** The OOP and the corresponding **b.** IP PFM images. **c.** The distribution of domain walls in IP PFM images. Black, brown and grey dotted lines correspond to dark, purple and bright contrasts in the OP image, respectively. Sky-blue lines is the non-neutral domain wall.

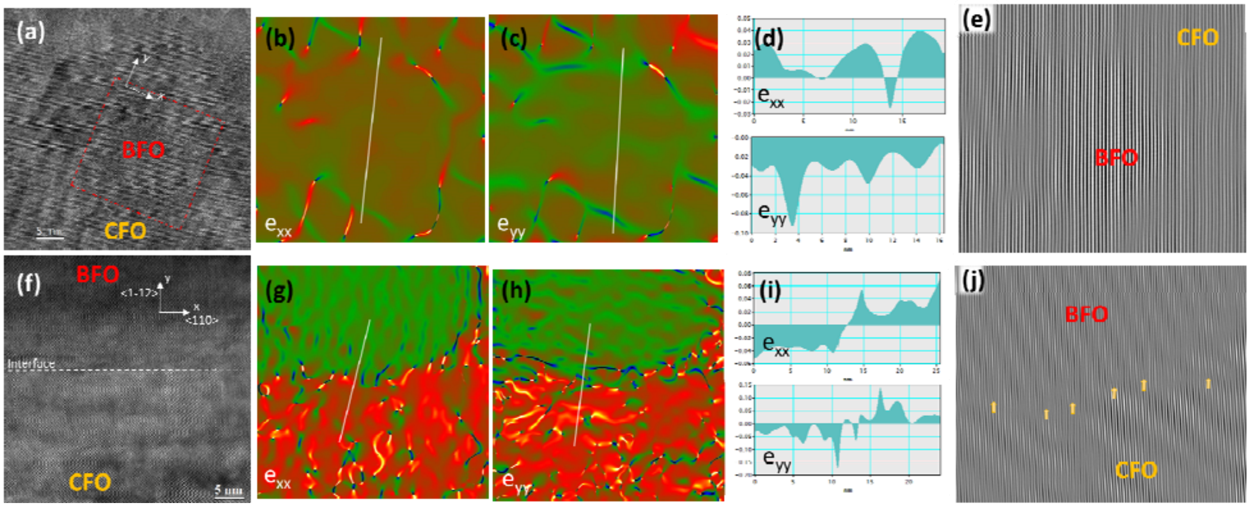

Supplementary Figure 6 Different strain distribution with different size of BFO mesocrystal. The TEM results of different size of BFO nanocrystal with **a-e.** ~15 nm and **f-j.** 170 nm in the sample of thickness 120nm, respectively. **a** and **f** The TEM images; **b**, **c**, **g** and **h** the corresponding strain mappings (e_{xx} and e_{yy}) of the TEM images in **a** and **f**; **d** and **i** the corresponding line scan of the lattice misfit of the white lines in **b**, **c**, **g** and **h**; **e** and **j** the Fourier filtered images.

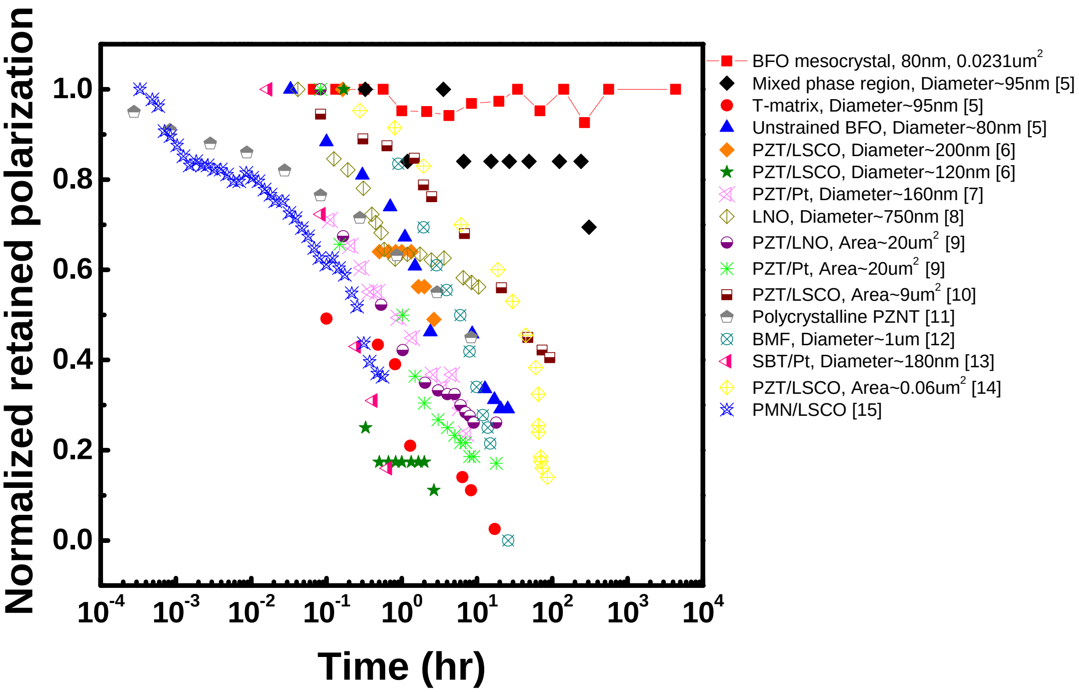

Supplementary Figure 7 The normalized retained polarization versus time. Comparison of normalized retained polarization versus the relaxing time between BFO mesocrystal and other ferroelectrics in earlier reports[5-15].

Supplementary Note 1 The observation of an intermediate state by PFM

In order to check the intermediate state during the 180° switching in BFO (111) mesocrystal, we have conducted PFM to switch the ferroelectricity of BFO nanocrystals as a function of voltage pulse duration. The OOP and IP phase images before and after the application of voltage pulse are shown in Supplementary Fig. 2a. For the analysis of polarization direction and switching modes, the OOP and IP phase images in Supplementary Fig. 2a are combined and the results are presented as the schematics shown in Supplementary Fig. 2b. Here, we compared the change of polarization in the white box as shown in Supplementary Fig. 2b and found the existence of the 71° and 109° switching events, delivering more evidence on the intermediate steps during the switching. However, if a large voltage with long pulse is applied, BFO nanocrystals are fully 180^0 switched, which is the initial state for our retention study.

Supplementary Note 2 The deviation of the hysteresis of BFO mesocrystal

Although the 40 nm thick system does show a larger coercive field, the deviation of the center of the hysteresis loop from the origin is also larger compared to the other two systems as shown in Supplementary Fig. 3. The deviation is considered as a driving force of the ferroelectric relaxation since it suggests an existence of the depolarization field.

Supplementary Note 3 The distribution of domain wall during the relaxation

Comparing the contrast of OOP and IP PFM image, there exist 71°, 109°, and non-neutral domain walls during the relaxation process as shown in Supplementary Fig. 4.

Therefore, additional experiments have been conducted. We traced the change of the polarization in BFO nanocrystals during the relaxation by PFM as shown in Supplementary Fig. 5. After the switching, the contrast in OOP PFM is bright and the one in IP PFM is brown, meaning the polarization points upwards. Then the polarization starts to reverse back as time

passes. During the relaxation, it shows a multi-step switching process since the contrast in both IP and OOP PFM images presents three colors as we mentioned in the manuscript. Based on a comparison of the OOP and IP PFM images, we can figure out the types of domain walls. From the observation, the area with the 71° and 109° domain wall has a large chance to reverse the polarization back. This could be attributed to the larger elastic energy in the vicinity of these ferroelastic domain walls. Therefore, the appearance of these domain walls cannot help to stabilize the polarization retention. All these new results are included here.

Supplementary Note 4 Strain analysis via TEM

The strain is imposed by the CFO matrix, so it is more important to compare the in-plane strain state. In order to realize this, we have carried out additional TEM and Geometric Phase Analysis (GPA)[1] analysis on the BFO nanocrystals with different sizes. Supplementary Figs. 6a and 6f are the plane-view HRTEM images of a 120 nm-thick BFO mesocrystal with different lateral size. The GPA[1] method was carried out based on the HRTEM images at the heterogeneous interfaces, which is an image processing technology used for mapping lattice displacement and has been successfully applied to characterize interface or defect structures such as misfit dislocations and their associated strain fields. The GPA strain maps of the BFO-CFO heterointerface shown in Supplementary Figs. 6b, 6c, 6f, and 6g were performed from the interface between ~15 nm, ~170 nm BFO nanocrystals and CFO matrix, respectively. For the e_{xx}, e_{yy} distortion maps (<110>, <1-12> directions) of small BFO nanocrystal (~15 nm) as shown in Supplementary Figs. 6b and 6c, the relative uniform color contrast was revealed with a lattice misfit of ~2.0% in e_{xx} (Supplementary Fig. 6d) and ~2.5% in e_{yy} (Supplementary Fig. 6d) between the CFO and BFO phases. This suggests that the BFO nanocrystal is constrained by a strong strain from the matrix. While for the distortion maps (e_{xx}, e_{yy}) of large BFO nanocrystal

(~170 nm) as shown in Supplementary Figs. 6g and 6h, the relative obvious color contrast were revealed with a lattice misfit of ~6.0% in e_{xx} and e_{yy} maps (Supplementary Fig. 6i) between the CFO and BFO, close to the calculated misfit using their bulk values. This demonstrates that the BFO-CFO lattices are almost relaxed. In addition, dislocations can be found in the interface between CFO matrix and large BFO nanocrystals (Supplementary Fig. 6j), suggesting the strain relaxation is mediated by the formation of dislocations, which cannot be found in the vicinity of small BFO nanocrystals (Supplementary Fig. 6e). This explains why large BFO nanocrystals relax much faster than small ones.

Supplementary Note 5 Comparison of the retained polarization among BFO mesocrystal and other ferroelectrics

In principle, there is no ferroelectric retention problem if appropriate electrical boundary conditions can be implemented. In a typical metal-ferroelectric-metal capacitor, the problem can be solved when a structure of symmetric electrodes is used[2]. However, there are several other configurations for practical applications, such as metal-ferroelectric-semiconductor and AFM tip/ferroelectric/metal, in which the symmetric electrodes cannot be made. Therefore, a severe retention problem occurs. We have tracked this problem on BFO for a while[3-5] and a serious retention problem can be observed on BFO films with various orientations. Supplementary Fig. 7 shows the comparison on the ferroelectric retention of BFO films, our BFO mesocrystal, and other ferroelectric materials with asymmetric electrical boundaries, suggesting that the ferroelectric retention is a generic problem. A new mechanism should be incorporated to provide a solution to this problem. This sets the novelty of this work. We provide a possible solution to this long-term issue, which hinders the applications of ferroelectrics in the past and multiferroics in the future. The relaxation of ferroelectric polarization follows certain physical principles.

Typically, the characteristic time of relaxation can be extracted based on the initial relaxation trend via various theoretical model. However, in our system, it doesn't show any degradation. Thus, we can say the retention time is much longer than our measuring period, one order longer at least. We call it "permanent", because it can last longer than the regular device life.

Supplementary References

1. Hÿtcha, M. J., Snoeckb, E. & Kilaasc, R. Quantitative measurement of displacement and strain fields from HREM micrographs. *Ultramicroscopy* **74**, 131-146 (1998).

2. Chu, Y. H. *et al.* Low voltage performance of epitaxial $BiFeO_3$ films on Si substrates through lanthanum substitution. *Appl. Phys. Lett.* **92,** 102909 (2008).

3. Chen, Y.-C., Lin, Q.-R. & Chu, Y.-H. Domain growth dynamics in single-domain-like thin films. *Appl. Phys. Lett.* **94,** 122908 (2009).

4. Chen, Y.-C., Ko, C.-H., Huang, Y.-C., Yang, J.-C. & Chu, Y.-H. Domain relaxation dynamics in epitaxial $BiFeO_3$ films: Role of surface charges. *J. Appl. Phys.* **112,** 052017 (2012).

5. Huang, Y.-C. *et al.* Giant Enhancement of Ferroelectric Retention in $BiFeO_3$ Mixed-Phase Boundary. *Adv. Mater.* **26,** 6335–6340 (2014).

6. Song, T. K., Yoon, J. G. & Kwun, S. I. Microscopic Polarization Retention Properties of Ferroelectric $Pb(Zr,Ti)O_3$ Thin Films. *Ferroelectrics* **335,** 61-68 (2006).

7. Fu, D. S., Suzuki, K., Kato, K. & Suzuki, H. Dynamics of nanoscale polarization backswitching in tetragonal lead zirconate titanate thin film. *Appl. Phys. Lett.* **82,** 2130 (2003).

8. Kan, Y. *et al.* Critical radii of ferroelectric domains for different decay processes in $LiNbO_3$ crystals. *Appl. Phys. Lett.* **91,** 132902 (2007).

9. Hong, J. W. *et al.* Nanoscale investigation of domain retention in preferentially oriented $PbZr_{0.53}Ti_{0.47}O_3$ thin films on Pt and on $LaNiO_3$. *Appl. Phys. Lett.* **75,** 3183 (1999).

10. Ganpule, C. S. *et al.* Domain nucleation and relaxation kinetics in ferroelectric thin films. *Appl. Phys. Lett.* **77,** 3275 (2000).

11. Gruverman, A. *et al.* Nanoscale imaging of domain dynamics and retention in ferroelectric thin films. *Appl. Phys. Lett.* **71,** 3492 (1997).

12. Zeng, H. R., Shimamura, K., Villora, E. G., Takekawa, S. & Kitamura, K. Domain growth kinetics and wall strain behavior in $BaMgF_4$ ferroelectric crystal by piezoresponse force microscopy. *J. Appl. Phys.* **101,** 074109 (2007).

13. Gruverman, A. & Tanaka, M. Polarization retention in $SrBi_2Ta_2O_9$ thin films investigated at nanoscale. *J. Appl. Phys.* **89,** 1836 (2001).

14. Ganpule, C. S. *et al.* Polarization relaxation kinetics and 180° domain wall dynamics in ferroelectric thin films. *Phys. Rev. B* **65,** 014101 (2001).

15. Shvartsman, V. V., Kholkin, A. L., Tyunina, M. & Levoska, J. Relaxation of induced polar state in relaxor $PbMg_{1/3}Nb_{2/3}O_3$ thin films studied by piezoresponse force microscopy. *Appl. Phys. Lett.* **86,** 222907 (2005).
